# Redetermination of the hexa­gonal struvite analogue Cs[Mg(OH_2_)_6_](PO_4_)

**DOI:** 10.1107/S1600536808023283

**Published:** 2008-07-31

**Authors:** Matthias Weil

**Affiliations:** a Institute for Chemical Technologies and Analytics, Division of Structural Chemistry, Vienna University of Technology, Getreidemarkt 9/164-SC, A-1060 Vienna, Austria

## Abstract

The structure of the hexa­gonal modification of caesium hexa­aqua­magnesium phosphate has been redetermined from single-crystal X-ray data. The previous refinement from photographic data [Ferrari, Calvaca & Nardelli (1955 ▶). *Gazz. Chim. Ital.* **85**, 1232–1238] was basically confirmed, but with all H atoms located and with all non H-atoms refined with anisotropic displacement parameters. The structure can be derived from the NiAs structure type: the PO_4_ tetra­hedra (3*m*. symmetry) are on the Ni positions and the complex [Mg(OH_2_)_6_] octa­hedra (3*m*. symmetry) are on the As positions. The building units are connected to each other by hydrogen bonds. The Cs^+^ cations (3*m*. symmetry) are located in the voids of this arrangement and exhibit a distorted cubocta­hedral 12-coordination by the O atoms of the water mol­ecules.

## Related literature

The crystal structure of struvite, NH_4_[Mg(OH_2_)_6_](PO_4_), was reported by Whitaker & Jeffery (1970*a* ▶,*b* ▶). Structure determinations of the hexa­gonal and cubic forms of Cs[Mg(OH_2_)_6_](PO_4_) were performed by Ferrari *et al.* (1955 ▶) and Massa *et al.* (2003 ▶), respectively. Crystal growth of struvite-like compounds using the gel diffusion technique was reported by Banks *et al.* (1975 ▶). For general background, see: Flack (1983 ▶).

## Experimental

### 

#### Crystal data

Cs[Mg(H_2_O)_6_](PO_4_)
*M* *_r_* = 360.29
Hexagonal, 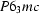

*a* = 6.8827 (8) Å
*c* = 11.9188 (16) Å
*V* = 488.97 (10) Å^3^
*Z* = 2
Mo *K*α radiation
μ = 4.04 mm^−1^
*T* = 293 (2) K
0.46 × 0.38 × 0.38 mm

#### Data collection

Stoe IPDS diffractometer
Absorption correction: numerical (*HABITUS*; Herrendorf, 1997 ▶) *T*_min_ = 0.213, *T*_max_ = 0.327
5412 measured reflections
368 independent reflections
367 reflections with *I* > 2σ(*I*)
*R*_int_ = 0.025

#### Refinement

*R*[*F* ^2^ > 2σ(*F* ^2^)] = 0.016
*wR*(*F* ^2^) = 0.034
*S* = 1.29
368 reflections
39 parameters
4 restraints
H atoms treated by a mixture of independent and constrained refinement
Δρ_max_ = 0.40 e Å^−3^
Δρ_min_ = −0.31 e Å^−3^
Absolute structure: Flack (1983 ▶), with 164 Friedel pairs
Flack parameter: −0.01 (3)

### 

Data collection: *EXPOSE* in *IPDS Software* (Stoe & Cie, 1998 ▶); cell refinement: *CELL* in *IPDS Software*; data reduction: *INTEGRATE* in *IPDS Software*; program(s) used to solve structure: *SHELXS97* (Sheldrick, 2008 ▶); program(s) used to refine structure: *SHELXL97* (Sheldrick, 2008 ▶); molecular graphics: *ATOMS* (Dowty, 2004 ▶); software used to prepare material for publication: *SHELXL97*.

## Supplementary Material

Crystal structure: contains datablocks I, global. DOI: 10.1107/S1600536808023283/br2080sup1.cif

Structure factors: contains datablocks I. DOI: 10.1107/S1600536808023283/br2080Isup2.hkl

Additional supplementary materials:  crystallographic information; 3D view; checkCIF report

## Figures and Tables

**Table 1:** Selected bond lengths (Å)

Cs—O3^i^	3.469 (5)
Cs—O4^i^	3.470 (4)
Cs—O4^ii^	3.742 (4)
Mg—O3	2.054 (4)
Mg—O4	2.082 (3)
P—O1	1.536 (3)
P—O2	1.539 (6)

Symmetry codes: (i) 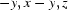; (ii) 
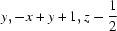
.

**Table 2:** Hydrogen-bond geometry (Å, °)

*D*—H⋯*A*	*D*—H	H⋯*A*	*D*⋯*A*	*D*—H⋯*A*
O3—H1⋯O2	0.93 (4)	1.71 (4)	2.643 (5)	180 (6)
O3—H2⋯O1^iii^	0.88 (4)	1.76 (5)	2.630 (5)	168 (7)
O4—H3⋯O1^iv^	0.83 (4)	1.85 (4)	2.672 (3)	177 (8)

Symmetry codes: (iii) 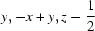; (iv) 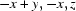.

**Fig. 1.**
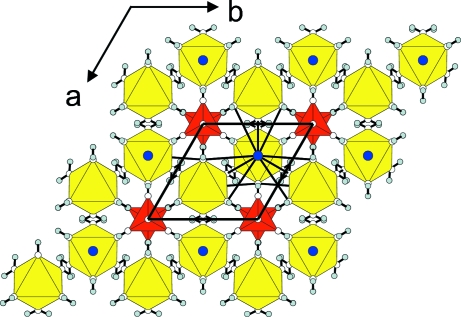
Projection of the crystal structure of Cs[Mg(H2O)6](PO4) along [001]. [Mg(OH2)6] octahedra are yellow, PO4 tetrahedra are red, Cs atoms are blue, O atoms are white and H atoms are grey. For one of the Cs+ cations the Cs—O bonds are indicated.

**Fig. 2.**
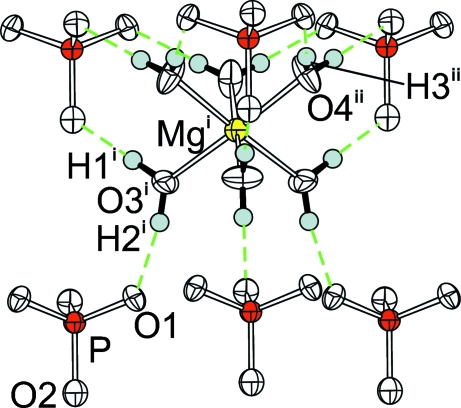
The [Mg(OH2)6] octahedron with six surrounding PO4 tetrahedra emphasizing the hydrogen bonding scheme (green dashed lines). The anisotropic displacement parameters are given at the 74% probability level. H atoms are given as spheres of arbitrary radius. [Symmetry codes: (i) y, x, z+1/2; (ii) -x+1, -y+1, -z+1.]

## supplementary crystallographic information

### Comment

Numerous compounds with general formula *A*[*B*(OH_2_)_6_]*X*O_4_, where *A* = alkali metal, NH_4_ or Tl, *B* = Mg or first row transition metal, and *X* = P or As, are known to crystallize in the orthorhombic struvite (NH_4_[Mg(OH_2_)_6_](PO_4_)) structure in space group *Pmn*2_1_ (Whitaker & Jeffery, 1979*a*,*b*). However, for the isoformular compounds Cs[Mg(OH_2_)_6_](*X*O_4_), where *X* = P and As, cubic and hexagonal forms were reported and structurally characterized (Ferrari *et al.*, 1955; Massa *et al.*, 2003). The cubic polymorph forms under hydrothermal conditions, whereas the hexagonal form is obtained under normal pressure and temperatures. All these structures can be described in terms of closed-packed layers with different stacking sequences (Massa *et al.*, 2003).

In this communication the redetermination of hexagonal Cs[Mg(OH_2_)_6_](PO_4_) is reported. The previous refinement from photographic data (Ferrari *et al.*, 1955) was basically confirmed, but with all H atoms located and with all non H-atoms refined with anisotropic displacement parameters.

In addition to the description of the hexagonal Cs[Mg(OH_2_)_6_](PO_4_) structure in terms of closed-packed layers (Massa *et al.*, 2003), the structure can be described as a derivative of the NiAs structure type. The centres of the slightly distorted PO_4_ tetrahedra (3*m*. symmetry) are situated on the Ni positions, whereas the centres of the likewise slightly distorted complex [Mg(H_2_O)_6_] octahedra (3*m*. symmetry) are situated on the As positions (Fig. 1). Thus one [Mg(H_2_O)_6_] octahedron is surrounded by six PO_4_ tetrahedra in a distorted trigonal–prismatic arrangement, whereas one PO_4_ tetrahedron is surrounded by six [Mg(H_2_O)_6_] octahedra in a distorted octahedral arrangement. The corresponding P—O and Mg—O distances are in the normal range (Table 1). These building units are linked *via* medium-strong hydrogen bonds (Table 2, Fig. 2). Details and differences of the hydrogen bonding schemes in cubic, hexagonal and struvite-type structures were discussed by Massa *et al.* (2003). The Cs^+^ cations are located in the voids of this arrangement and exhibit a distorted cuboctahedral 12-coordination [9+3] to the oxygen atoms of the water molecules (Table 1).

### Experimental

Colourless crystals of Cs[Mg(OH_2_)_6_](PO_4_) with an edge-length up to 2 mm and mostly spherical habit were grown by means of the gel diffusion technique, following a slightly modified procedure as that given by Banks *et al.* (1975). Aqueous solutions of 0.025 *M* MgSO_4_ and 0.02 *M* Na_4_edta (edta = ethylenediaminetetraacetate) were adjusted to pH 10 with NaOH. Commercially available gelatine foils (5 g) were dissolved in the hot resulting 100 ml solution and allowed to form a gel inside a large test tube overnight. When the gel had set, an equivalent amount of a solution of 0.025 *M* CsH_2_PO_4_ (50 ml) was carefully poured over the gel. This solution was then adjusted to pH 8.5 with NaOH. The test tube was covered with parafilm and the crystal growth proceeded at the gel–liquid interface and into the gel. Crystals large enough for conventional X-ray analysis grew within one week at room temperature. They were separated mechanically from the gel and were washed with a water/ethanol/acetone (1/3/1) mixture.

### Refinement

The positions of the H atoms were found from difference Fourier maps and were refined with soft distance restraints (*d*(O—H) = 0.90 (5) Å) and a common *U*_iso_ parameter.

### Crystal data

**Table:**

Cs[Mg(H_2_O_1_)_6_](PO_4_)	*Z* = 2
*M**_r_* = 360.29	*F*_000_ = 348
Hexagonal, *P*6_3_*m**c*	*D*_x_ = 2.447 Mg m^−^^3^
Hall symbol: P 6c -2c	Mo *K*α radiation λ = 0.71073 Å
*a* = 6.8827 (8) Å	Cell parameters from 1544 reflections
*b* = 6.8827 (8) Å	θ = 3.4–25.9º
*c* = 11.9188 (16) Å	µ = 4.04 mm^−^^1^
α = 90º	*T* = 293 (2) K
β = 90º	Block, colourless
γ = 120º	0.46 × 0.38 × 0.38 mm
*V* = 488.97 (10) Å^3^	

### Data collection

**Table:**

Stoe IPDS diffractometer	368 independent reflections
Radiation source: fine-focus sealed tube	367 reflections with *I* > 2σ(*I*)
Monochromator: graphite	*R*_int_ = 0.025
*T* = 293(2) K	θ_max_ = 25.7º
ω scans	θ_min_ = 3.4º
Absorption correction: numerical(HABITUS; Herrendorf, 1997)	*h* = −8→8
*T*_min_ = 0.213, *T*_max_ = 0.327	*k* = −8→8
5412 measured reflections	*l* = −12→13

### Refinement

**Table:**

Refinement on *F*^2^	Hydrogen site location: difference Fourier map
Least-squares matrix: full	H atoms treated by a mixture of independent and constrained refinement
*R*[*F*^2^ > 2σ(*F*^2^)] = 0.016	*w* = 1/[σ^2^(*F*_o_^2^) + (0.0029*P*)^2^ + 0.8141*P*] where *P* = (*F*_o_^2^ + 2*F*_c_^2^)/3
*wR*(*F*^2^) = 0.034	(Δ/σ)_max_ < 0.001
*S* = 1.29	Δρ_max_ = 0.40 e Å^−^^3^
368 reflections	Δρ_min_ = −0.31 e Å^−^^3^
39 parameters	Extinction correction: none
4 restraints	Absolute structure: Flack (1983), with 164 Friedel pairs
Primary atom site location: structure-invariant direct methods	Flack parameter: −0.01 (3)
Secondary atom site location: difference Fourier map	

### Special details

**Table:**

Geometry. All e.s.d.'s (except the e.s.d. in the dihedral angle between two l.s. planes) are estimated using the full covariance matrix. The cell e.s.d.'s are taken into account individually in the estimation of e.s.d.'s in distances, angles and torsion angles; correlations between e.s.d.'s in cell parameters are only used when they are defined by crystal symmetry. An approximate (isotropic) treatment of cell e.s.d.'s is used for estimating e.s.d.'s involving l.s. planes.
Refinement. Refinement of *F*^2^ against ALL reflections. The weighted *R*-factor *wR* and goodness of fit *S* are based on *F*^2^, conventional *R*-factors *R* are based on *F*, with *F* set to zero for negative *F*^2^. The threshold expression of *F*^2^ > σ(*F*^2^) is used only for calculating *R*-factors(gt) *etc*. and is not relevant to the choice of reflections for refinement. *R*-factors based on *F*^2^ are statistically about twice as large as those based on *F*, and *R*- factors based on ALL data will be even larger.

### Fractional atomic coordinates and isotropic or equivalent isotropic displacement parameters (Å^2^)

**Table:**

	*x*	*y*	*z*	*U*_iso_*/*U*_eq_	
Cs	0.3333	0.6667	0.02181 (4)	0.03126 (15)	
Mg	0.6667	0.3333	0.1436 (2)	0.0159 (5)	
P	0.0000	0.0000	0.29985 (14)	0.0137 (5)	
O1	0.1220 (2)	0.2440 (4)	0.3413 (3)	0.0173 (6)	
O2	0.0000	0.0000	0.1707 (5)	0.0189 (13)	
O3	0.3747 (6)	0.1873 (3)	0.0521 (3)	0.0265 (9)	
O4	0.5229 (3)	0.0458 (5)	0.2427 (3)	0.0282 (8)	
H1	0.243 (8)	0.122 (4)	0.094 (5)	0.048 (9)*	
H2	0.350 (12)	0.175 (6)	−0.021 (4)	0.048 (9)*	
H3	0.397 (8)	−0.010 (14)	0.272 (5)	0.048 (9)*	

### Atomic displacement parameters (Å^2^)

**Table:**

	*U*^11^	*U*^22^	*U*^33^	*U*^12^	*U*^13^	*U*^23^
Cs	0.02671 (16)	0.02671 (16)	0.0404 (3)	0.01335 (8)	0.000	0.000
Mg	0.0158 (8)	0.0158 (8)	0.0160 (16)	0.0079 (4)	0.000	0.000
P	0.0139 (5)	0.0139 (5)	0.0134 (13)	0.0069 (2)	0.000	0.000
O1	0.0172 (9)	0.0139 (13)	0.0195 (17)	0.0070 (7)	−0.0023 (6)	−0.0046 (12)
O2	0.0177 (19)	0.0177 (19)	0.021 (4)	0.0089 (10)	0.000	0.000
O3	0.0142 (14)	0.0406 (14)	0.016 (3)	0.0071 (7)	−0.0023 (11)	−0.0012 (5)
O4	0.0191 (11)	0.0252 (19)	0.042 (2)	0.0126 (10)	0.0096 (7)	0.0191 (15)

### Geometric parameters (Å, °)

**Table:**

Cs—O3^i^	3.469 (5)	Mg—O3	2.054 (4)
Cs—O3^ii^	3.469 (5)	Mg—O3^v^	2.054 (4)
Cs—O3^iii^	3.469 (5)	Mg—O4^ix^	2.082 (3)
Cs—O3^iv^	3.469 (5)	Mg—O4	2.082 (3)
Cs—O3^v^	3.469 (5)	Mg—O4^v^	2.082 (3)
Cs—O3	3.469 (5)	Mg—Cs^x^	4.2306 (10)
Cs—O4^i^	3.470 (4)	Mg—Cs^xi^	4.2306 (10)
Cs—O4^iv^	3.470 (4)	Mg—Cs^xii^	4.508 (3)
Cs—O4^v^	3.470 (4)	P—O1	1.536 (3)
Cs—O4^vi^	3.742 (4)	P—O1^iv^	1.536 (3)
Cs—O4^vii^	3.742 (4)	P—O1^xiii^	1.536 (3)
Cs—O4^viii^	3.742 (4)	P—O2	1.539 (6)
Mg—O3^ix^	2.054 (4)		
			
O3^i^—Cs—O3^ii^	51.51 (11)	O3^v^—Cs—O4^vii^	97.18 (5)
O3^i^—Cs—O3^iii^	67.77 (11)	O3—Cs—O4^vii^	118.02 (7)
O3^ii^—Cs—O3^iii^	118.93 (2)	O4^i^—Cs—O4^vii^	112.101 (19)
O3^i^—Cs—O3^iv^	118.93 (2)	O4^iv^—Cs—O4^vii^	145.46 (3)
O3^ii^—Cs—O3^iv^	165.52 (11)	O4^v^—Cs—O4^vii^	145.46 (3)
O3^iii^—Cs—O3^iv^	51.51 (11)	O4^vi^—Cs—O4^vii^	46.74 (8)
O3^i^—Cs—O3^v^	118.93 (2)	O3^i^—Cs—O4^viii^	118.02 (7)
O3^ii^—Cs—O3^v^	67.77 (11)	O3^ii^—Cs—O4^viii^	97.18 (5)
O3^iii^—Cs—O3^v^	165.52 (11)	O3^iii^—Cs—O4^viii^	118.02 (7)
O3^iv^—Cs—O3^v^	118.93 (2)	O3^iv^—Cs—O4^viii^	97.18 (5)
O3^i^—Cs—O3	165.52 (11)	O3^v^—Cs—O4^viii^	71.50 (7)
O3^ii^—Cs—O3	118.93 (2)	O3—Cs—O4^viii^	71.50 (7)
O3^iii^—Cs—O3	118.93 (2)	O4^i^—Cs—O4^viii^	145.46 (3)
O3^iv^—Cs—O3	67.77 (11)	O4^iv^—Cs—O4^viii^	145.46 (3)
O3^v^—Cs—O3	51.51 (11)	O4^v^—Cs—O4^viii^	112.101 (19)
O3^i^—Cs—O4^i^	48.58 (7)	O4^vi^—Cs—O4^viii^	46.74 (8)
O3^ii^—Cs—O4^i^	48.58 (7)	O4^vii^—Cs—O4^viii^	46.74 (8)
O3^iii^—Cs—O4^i^	88.12 (6)	O3^ix^—Mg—O3	94.42 (16)
O3^iv^—Cs—O4^i^	117.22 (7)	O3^ix^—Mg—O3^v^	94.42 (16)
O3^v^—Cs—O4^i^	88.12 (6)	O3—Mg—O3^v^	94.42 (16)
O3—Cs—O4^i^	117.22 (7)	O3^ix^—Mg—O4^ix^	87.27 (10)
O3^i^—Cs—O4^iv^	88.12 (6)	O3—Mg—O4^ix^	177.5 (2)
O3^ii^—Cs—O4^iv^	117.22 (7)	O3^v^—Mg—O4^ix^	87.27 (10)
O3^iii^—Cs—O4^iv^	48.58 (7)	O3^ix^—Mg—O4	87.27 (10)
O3^iv^—Cs—O4^iv^	48.58 (7)	O3—Mg—O4	87.27 (10)
O3^v^—Cs—O4^iv^	117.22 (7)	O3^v^—Mg—O4	177.5 (2)
O3—Cs—O4^iv^	88.12 (6)	O4^ix^—Mg—O4	90.98 (19)
O4^i^—Cs—O4^iv^	68.67 (8)	O3^ix^—Mg—O4^v^	177.5 (2)
O3^i^—Cs—O4^v^	117.22 (7)	O3—Mg—O4^v^	87.27 (10)
O3^ii^—Cs—O4^v^	88.12 (6)	O3^v^—Mg—O4^v^	87.27 (10)
O3^iii^—Cs—O4^v^	117.22 (7)	O4^ix^—Mg—O4^v^	90.98 (19)
O3^iv^—Cs—O4^v^	88.12 (6)	O4—Mg—O4^v^	90.98 (19)
O3^v^—Cs—O4^v^	48.58 (7)	O1—P—O1^iv^	110.17 (14)
O3—Cs—O4^v^	48.58 (7)	O1—P—O1^xiii^	110.17 (14)
O4^i^—Cs—O4^v^	68.67 (8)	O1^iv^—P—O1^xiii^	110.17 (14)
O4^iv^—Cs—O4^v^	68.67 (8)	O1—P—O2	108.76 (14)
O3^i^—Cs—O4^vi^	97.18 (5)	O1^iv^—P—O2	108.76 (14)
O3^ii^—Cs—O4^vi^	118.02 (7)	O1^xiii^—P—O2	108.76 (14)
O3^iii^—Cs—O4^vi^	71.50 (7)	Mg—O3—Cs^x^	96.63 (6)
O3^iv^—Cs—O4^vi^	71.50 (7)	Mg—O3—Cs	96.63 (6)
O3^v^—Cs—O4^vi^	118.02 (7)	Cs^x^—O3—Cs	165.52 (11)
O3—Cs—O4^vi^	97.18 (5)	Mg—O3—H1	116 (4)
O4^i^—Cs—O4^vi^	145.46 (3)	Mg—O3—H2	132 (5)
O4^iv^—Cs—O4^vi^	112.101 (19)	H1—O3—H2	113 (6)
O4^v^—Cs—O4^vi^	145.46 (3)	Mg—O4—Cs^x^	96.07 (16)
O3^i^—Cs—O4^vii^	71.50 (7)	Mg—O4—Cs^xii^	97.31 (14)
O3^ii^—Cs—O4^vii^	71.50 (7)	Cs^x^—O4—Cs^xii^	166.63 (10)
O3^iii^—Cs—O4^vii^	97.18 (5)	Mg—O4—H3	125 (4)
O3^iv^—Cs—O4^vii^	118.02 (7)		

Symmetry codes: (i) *x*, *y*+1, *z*; (ii) −*y*+1, *x*−*y*+1, *z*; (iii) −*x*+*y*, −*x*+1, *z*; (iv) −*y*, *x*−*y*, *z*; (v) −*x*+*y*+1, −*x*+1, *z*; (vi) *y*, −*x*+*y*+1, *z*−1/2; (vii) −*x*+1, −*y*+1, *z*−1/2; (viii) *x*−*y*, *x*, *z*−1/2; (ix) −*y*+1, *x*−*y*, *z*; (x) *x*, *y*−1, *z*; (xi) *x*+1, *y*, *z*; (xii) *x*−*y*+1, *x*, *z*+1/2; (xiii) −*x*+*y*, −*x*, *z*.

### Hydrogen-bond geometry (Å, °)

**Table:**

*D*—H···*A*	*D*—H	H···*A*	*D*···*A*	*D*—H···*A*
O3—H1···O2	0.93 (4)	1.71 (4)	2.643 (5)	180 (6)
O3—H2···O1^xiv^	0.88 (4)	1.76 (5)	2.630 (5)	168 (7)
O4—H3···O1^xiii^	0.83 (4)	1.85 (4)	2.672 (3)	177 (8)

Symmetry codes: (xiv) *y*, −*x*+*y*, *z*−1/2; (xiii) −*x*+*y*, −*x*, *z*.
